# Process Variability—Technological Challenge and Design Issue for Nanoscale Devices

**DOI:** 10.3390/mi10010006

**Published:** 2018-12-23

**Authors:** Jürgen Lorenz, Eberhard Bär, Sylvain Barraud, Andrew R. Brown, Peter Evanschitzky, Fabian Klüpfel, Liping Wang

**Affiliations:** 1 Fraunhofer Institut für Integrierte Systeme und Bauelementetechnologie, Schottkystrasse 10, 91058 Erlangen, Germany; eberhard.baer@iisb.fraunhofer.de (E.B.); peter.evanschitzky@iisb.fraunhofer.de (P.E.); Fabian.Kluepfel@iisb.fraunhofer.de (F.K.); 2 CEA, LETI, MINATEC campus and Univ. Grenoble Alpes, 38054 Grenoble, France; sylvain.barraud@cea.fr; 3 Synopsys Northern Europe Ltd., Glasgow G3 8HB, UK; Andrew.Brown@synopsys.com (A.R.B.); Liping.Wang@synopsys.com (L.W.)

**Keywords:** process simulation, device simulation, compact models, process variations, systematic variations, statistical variations, FinFETs, nanowires, nanosheets

## Abstract

Current advanced transistor architectures, such as FinFETs and (stacked) nanowires and nanosheets, employ truly three-dimensional architectures. Already for aggressively scaled bulk transistors, both statistical and systematic process variations have critically influenced device and circuit performance. Three-dimensional device architectures make the control and optimization of the device geometries even more important, both in view of the nominal electrical performance to be achieved and its variations. In turn, it is essential to accurately simulate the device geometry and its impact on the device properties, including the effect caused by non-idealized processes which are subject to various kinds of systematic variations induced by process equipment. In this paper, the hierarchical simulation system developed in the SUPERAID7 project to study the impact of variations from equipment to circuit level is presented. The software system consists of a combination of existing commercial and newly developed tools. As the paper focuses on technological challenges, especially issues resulting from the structuring processes needed to generate the three-dimensional device architectures are discussed. The feasibility of a full simulation of the impact of relevant systematic and stochastic variations on advanced devices and circuits is demonstrated.

## 1. Introduction

**Figure 1:**
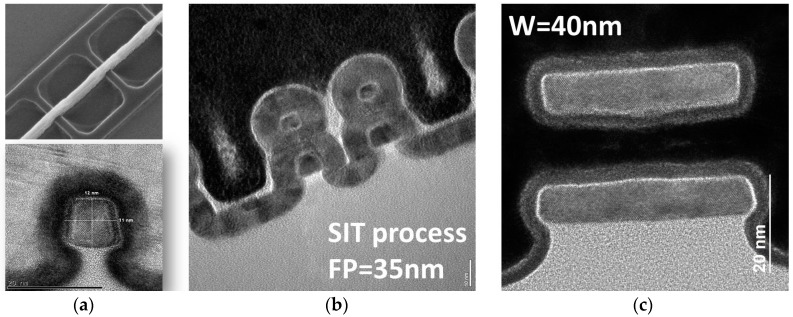
Example of transistor as addressed in SUPERAID7: (**a**) Trigate; (**b**) stacked nanowire; (**c**) stacked nanosheet.

Aggressively scaled transistors are affected by three kinds of process variations. Most frequently and since long discussed in the literature are statistical process variations which are caused by the granularity of matter, such as Random Dopant Fluctuations (RDF). However, as summarized earlier [1] a diversity of systematic process variations is caused by non-idealities of process equipment, like inhomogeneity of gas flow or temperature distributions, or imperfect control of parameters like the distance between the last lens and the wafer in lithography, the so-called defocus. Moreover, layout effects caused, e.g., by pattern density also affect the results of various process steps [1]. Especially for aggressively scaled three-dimensional devices such as FinFETs or nanowires as shown in Figure 1, not only systematic variations of simple geometrical parameters such as the gate length must be considered, but also three-dimensional shapes may vary, critically affecting device performance. These requirements are being addressed and met in the cooperative project SUPERAID7 [2] funded by the European Commission within the Horizon 2020 programme.

Whereas the impact of statistical variations such as Random Dopant Fluctuations (RDF), Metal Gate Granularity (MGG), and Line Edge Roughness (LER) have been frequently and since long discussed in the literature (e.g., in [3,4,5]), the effects of systematic process variations have so far got much less attention. Some publications with involvement of authors of this paper referred to bulk transistors [1,6,7,8,9]. Two examples for the impact of patterning processes on FinFET transistors were presented earlier [10,11,12], in the latter case also discussing the impact on a static random-access memory (SRAM) cell. However, these papers did not discuss the key differences between the different patterning processes in terms of variability and impact on design. Furthermore, the general case where not only two or three geometrical parameters of the transistor, e.g., fin width and gate length, are changed but also the shape of a fin or of nanowires was not considered

In the following, the main processes available for the patterning of three-dimensional nanoscale transistors are discussed concerning their simulation and their variability, and a method to extract compact models which include both systematic and stochastic variations which may also affect the shape of the transistor is presented.

## 2. Materials and Methods

This paper reports about results obtained in the cooperative project SUPERAID7 [2], funded within the Horizon 2020 programme of the European Union, and partly about related background work from Fraunhofer IISB, and from partners as cited. Within SUPERAID7 a software system for the simulation of the impact of systematic and statistical process variations on advanced More Moore devices and circuits down to the 7 nm node and below has been developed, including especially interconnects. Besides enhanced and new software tools this needs improved physical models and extended compact models. In terms of software integration and application, SUPERAID7 and this paper are partly based on the SENTAURUS simulation system from Synopsys [13], which is for academic use also available via EUROPRACTICE [14]. Topography simulation for this paper has been performed with the tools Dr.LiTHO [15], ANETCH [16], and DEP3D [17], which are available under license from Fraunhofer IISB, and also with ViennaTS which is available from TU Wien as Open Source [18].

## 3. Results

### 3.1. Nanoscale Patterning Processes and Their Variations

Besides the physical properties of the semiconductor material used, which among others limit carrier mobility, the patterning of the transistors has increasingly become challenging. This is both due to the ever-smaller feature sizes needed and the complex three-dimensional geometries employed for nanoscale transistors in order to achieve good channel control. In the following, this development is outlined especially in view of process variations and design implications.

#### 3.1.1. Lithography

**Figure 2:**
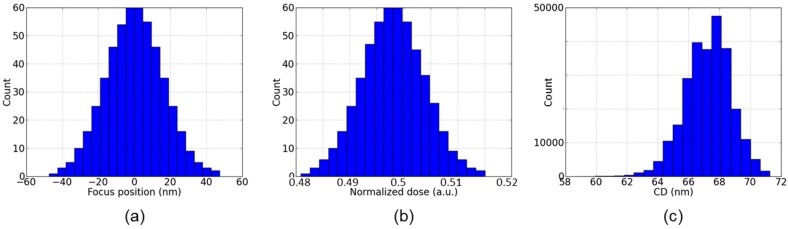
Impact of variations of focus and dose on a 193 nm dry lithography process with adapted illumination for 65 nm lines/130 nm spaces (duty factor 1:2): Variation of (**a**) focus and (**b**) threshold considered; (**c**) resulting variation of CD.

**Figure 3:**
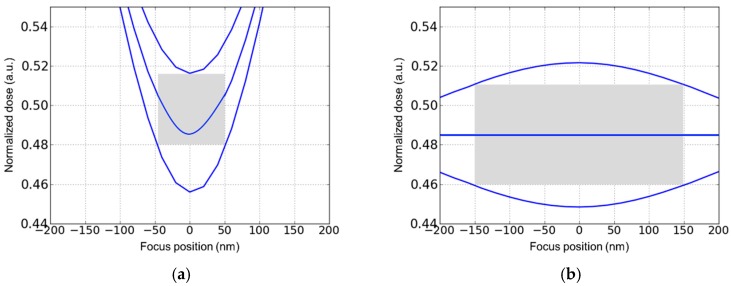
Aerial image-based process window on a 193 nm dry lithography system with adapted illumination as in Figure 2, for (**a**) 65 nm lines/130 nm spaces (duty factor 1:2). (**b**) 65 nm dense lines: 65nm lines and spaces with a duty factor of 1:1.

For traditional bulk CMOS, mainly gates and their spacers had to be patterned. In corresponding simulations of lithography, etching, and deposition mainly, the resulting footprint of gate and spacer was relevant, because this defined the physical gate length L and width W. In turn, variations in these patterning processes led to variations of L and W, which could readily be considered during the extraction of compact models [8]. In most cases, standard optical lithography was used, partly including water immersion [19]. Such lithography steps are subject to variations of the distance between the last lens of the optical system and the photoresist to be developed (which can be transferred into the so-called focus position or focus) and of the illumination dose used. These lead to variations of the feature size printed, the so-called critical dimensions (CD). Generally, best resolution is obtained for so-called dense lines, that is, a regular pattern of lines and spaces of the same width (duty factor 1:1). However, the situation encountered during circuit fabrication usually deviates from this situation. In Figure 2, the CD of the intensity distribution of the light just above the resist (the so-called aerial image) is shown for 65 nm lines and 130 nm spaces (duty factor 1:2). The figure illustrates how the variations of focus and dose lead to variations of the CD of the aerial image, which would then during resist development be transferred into variations of the CD of the resist structures. Moreover, the distribution of the CDs is shifted from the nominal 65 nm to larger values. Figure 3 shows the dependence of the CD on focus and dose: Here, the central curve shows the combinations of focus and dose which lead to the nominal CD of 65 nm. The two other curves show the combinations of focus and dose which result in a CD increased (upper line) or decreased (lower line) by 10%, respectively. A standard task in the lithography community is then to optimize the lithography process in terms of its stability against variations of dose and focus. Here, the so-called process window is maximized, which is the largest rectangle in the space of focus and dose which results in CDs which deviate not more than 10% from the targets values. With the duty factor of 1:2 shown in Figure 3a, the process window is rather small. For the ideal case of dense lines with a duty factor of 1:1, shown in Figure 3b, the process window is much larger. This illustrates that both the resolution and also the variability of a lithography process critically depend not only on the size but also on the layout of the features or circuits to be printed.

**Figure 4:**
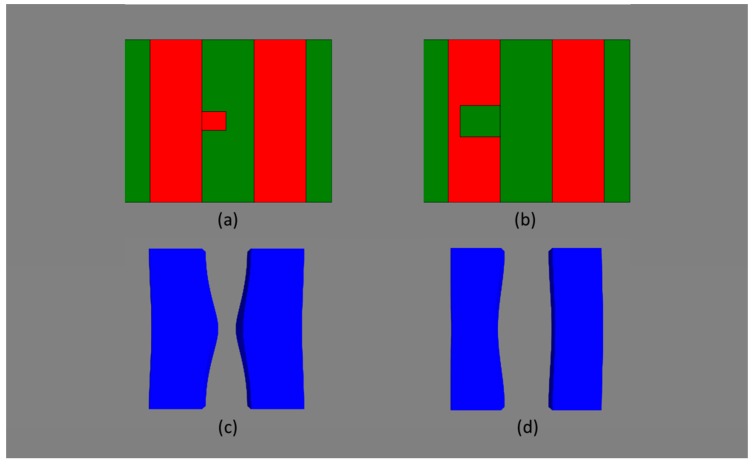
Example for defect printing in 193 nm dry optical lithography. Mask defects considered (65 nm dense lines/spaces with (**a**) 30 nm extrusion and (**b**) 50 nm intrusion; (**c**) and (**d**) resulting resist geometries for (**a**) and (**b**), respectively, for lithography with ideal focus and dose.

**Figure 5:**
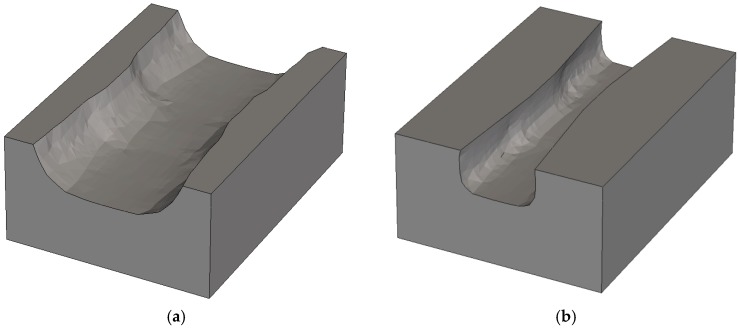
Simulation of oxide etching using the resist shape of Figure 4c as a mask. (**a**) Isotropic etching. (**b**) Anisotropic etching. To allow better visualization of the etched material, the resist mask is not shown. The lateral width of the simulation domain is 190 nm.

Another important non-ideality which challenges aggressive patterning steps is the printing of defects. These include especially defects in the multilayer mirrors used for masks and instead of lenses in an Extreme Ultraviolet (EUV) lithography step, or defects of the mask structures [20]. Equipment and mask makers generally try to compensate mirror defects [20]. Contamination with particles during the handling of masks can be compensated by the use of pellicles which move the particles out of the mask area and in turn make sure that they do not print [21]. The effect of defects of the mask itself should be considered as a process variation insofar as the patterns generated on the wafer may deviate from the shapes which would be generated without a defect. Figure 4 shows an example for mask defect printing as frequently simulated in the lithography community. However, the impact on device level has so far not been considered systematically. In order to study this effect, the subsequent etching of the underlying layer to be patterned by the lithography step must be considered. This needs the intimate coupling of lithography and etching simulation, which is one of the core topics of the SUPERAID7 project. Figure 5 shows how the image of the mask defect shown in Figure 4 is transferred into the underlying layer for the two extreme cases of anisotropic and isotropic etching. The etching simulator ANETCH [16] used for this example can be calibrated to the specific etching process applied and therefore enables the assessment of defect transfer to patterned layers.

Shrinking device dimensions have led to more complex device architectures in order to maintain sufficient electrostatic control of the channel, low leakage and high drive currents. In order to extend CMOS scaling to its limits, first buried oxide layers were introduced for the so-called SOI (silicon-on-insulator) transistors. This has been followed by transistor architectures such as FinFETs, (stacked) nanowires and (stacked) nanosheets. These small and truly three-dimensional structures lead to considerable challenges for the patterning processes and for their simulation. As outlined below, they also generate different problems in terms of variability and impact on design. The problem starts from the basic limitation of optical lithography, where the minimum half pitch printed is given by d = k_1_·λ/NA, where k_1_ is the technology factor with minimum value of 0.25 for dense lines, λ is the wavelength of the light used, and NA is the numerical aperture, equal to the sine of the opening angle of the last lens times the refractive index of the medium between lens and wafer (1 for air, and 1.44 for water). With 193 nm being the minimum wavelength at which lenses are sufficiently transparent, a minimum half pitch for dense lines of about 34 nm results in case of water immersion lithography.

**Figure 6:**
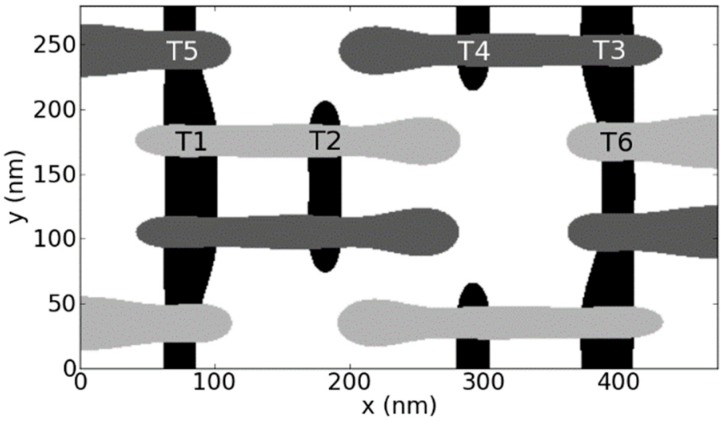
Top-view of lithography simulation of an SRAM cell. Black vertical features are the active silicon areas, the horizontal light grey and dark grey features are the polysilicon gate areas generated with the first and the second incremental lithography step, respectively. Transistors T1 to T6 are explained in the text.

The favorite industrial approaches for the patterning of nanoscale devices smaller than that limit are various kinds of double or even quadruple patterning. In Litho-Etch-Litho-Etch (LELE) [22] and Litho-Freeze-Litho-Etch (LFLE) [23] the number of features is doubled and the pitch size halved, by exposing the wafer twice with two different masks (e.g., the mask shifted by half the pitch for the second illumination), employing some memory process of the photoresist and finally developing the resist after the second illumination. Consequently, focus and especially dose variations are not correlated between these two incremental lithography steps. This has severe consequences on circuit level: e.g., for an SRAM cell the densest structures are the polysilicon gate lines, see Figure 6. As shown therein, the three transistors T1, T2, and T6 are patterned with one illumination step, whereas the transistors T3, T4, and T 5 are patterned with the other illumination step. In turn, focus and dose variations correlate within each of the two triples of transistors, but especially in terms of dose not between the two triples. This has severe consequences for variability-aware circuit simulation. Considering dose variations in the double patterning step for example, one value of the varying dose must be used for the first triple and a (likely different) value for the second triple. In turn, the dose variations may, e.g., cause an increase of the CD for the first triple and a decrease for the second one, leading to negative consequences for circuit performance. This was discussed in more detail elsewhere [10].

**Figure 7:**
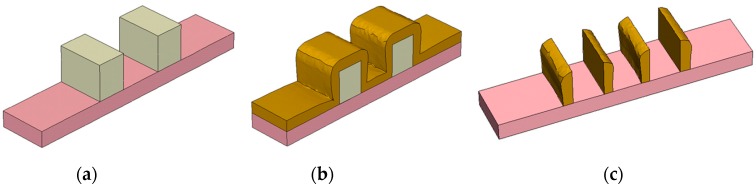
Example for the simulation of Self-Aligned Double Patterning: (**a**) carbon lines patterned by optical lithography; (**b**) layer deposition; (**c**) spacer pattern after back etching.

A different situation holds for Self-Aligned Double Patterning (SADP): Here, first a carbon layer is patterned by optical lithography. Then, spacers are created by deposition followed by back etching, see Figure 7. In result, the pattern density is doubled compared with the initial pattern of the carbon lines. The CDs of the final spacers are nearly independent of the lithography step, and are largely defined by the deposition and etching processes used. Variations of the CDs depend on the parameters of the etching and deposition processes [24]. Because etching and deposition partly depend on the open view angle of the surface element towards the reactor volume, a layout dependence of the CD is introduced: The CD of outer lines is different from the CD of inner lines. An important target of process optimization is the tuning of the deposition and etching processes in order to minimize their variability across the wafer and the layout impact explained above.

For current FinFET transistors, usually SADP is applied for the patterning of the fins as dense lines, whereas LELE is used for gate patterning, because it is more flexible in terms of the layouts.

**Figure 8:**
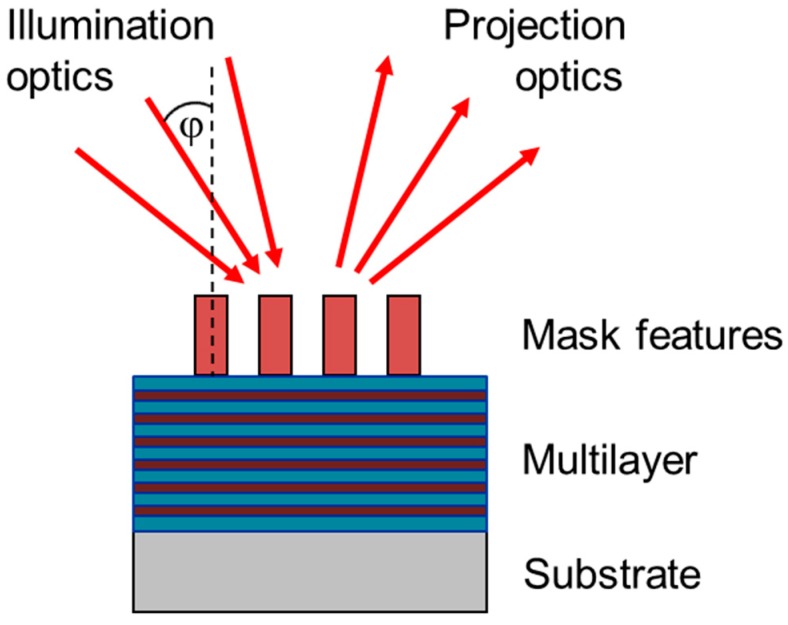
Principle of illumination of an EUV mirror and mask. ϕ denotes the chief ray angle.

Another design issue appears in case of EUV. Here, soft X-rays with 13.5 nm wavelength are used instead of laser light. Because lenses are not sufficiently transparent for light below 193 nm, in EUV instead of refractive optics, reflective optics are used, consisting of multilayer mirrors and new absorber materials [20]. Whereas dose and defocus continue to be major sources of systematic process variability, EUV leads to an additional design issue, which has so far not been addressed when discussing process flows for the fabrication of advanced transistors. Because the reflected light must be separated from the incoming light, illumination of all mirrors and especially of the mask is not vertical but tilted by a so-called “chief ray angle” of about 5.3°, see Figure 8. In turn, features generate an asymmetric shadowing relative to the plane of incidence, resulting in a position shift, a CD increase and a telecentricity error. The first two effects can be compensated by the so-called “Optical Proximity Correction” (OPC) techniques. The third effect introduces an additional source of variations since it causes a focus dependent position of the features. Furthermore, the absorber features are thick compared to the illumination wavelength resulting in phase deformations causing mask-induced aberration like effects. Finally, a new class of defects, the multilayer defects, introduce new issues in the field of defect detection and repair.

In summary, various kinds of systematic, layout-dependent and defect-induced variations influence the patterns generated in a lithography or multiple patterning process. There are major even qualitative differences in terms of variability between optical lithography, SADP and other double patterning processes. Etching and deposition, which are key components of SADP, are discussed in the next chapter.

#### 3.1.2. Deposition and Etching

In semiconductor technology, a large variety of deposition and etching processes is employed. Deposition processes differ among others in terms of the properties of the deposited layers and the layer conformality. The latter for instance critically influences the filling of contact holes and trenches. Etching processes differ, e.g., in terms of selectivity concerning the materials to be etched, etch-induced damage, and the degree of isotropy and anisotropy. Equipment simulation tools such as CFD-ACE+ [25] and Q-VT [26], which model the electrical, thermal, fluid-dynamical, and plasma properties of the reactor can be used to predict the fluxes and energies of different species (ions, neutrals) above the flat or patterned wafer. Using these as boundary conditions, feature-scale simulation allows one to simulate the evolving geometry during deposition or etching. To this end, the interaction of the different species with the substrate is considered and local values of the different fluxes are extracted which allow one to determine local etching or deposition rates. Examples for this approach are given in [27] for deposition and in [28] for etching. The discussion of the large variety of deposition and etching processes is beyond the scope of this paper.

Deposition and etching processes are subject to several sources of systematic variability. Depending on the process in question, these include the inhomogeneity of gas flow and temperature in the etching or deposition reactor, the non-homogeneous emission of metal atoms, e.g., from a sputter target, or the finite size of a sputter target. These sources lead to variations of deposition and etching rates across the wafer. Furthermore, non-vertical incidence of contributing species can lead to layout-dependent deposition or etching rates, as discussed above for the SADP process. An overview of sources of variability in deposition and etching processes is given elsewhere [1].

**Figure 9:**
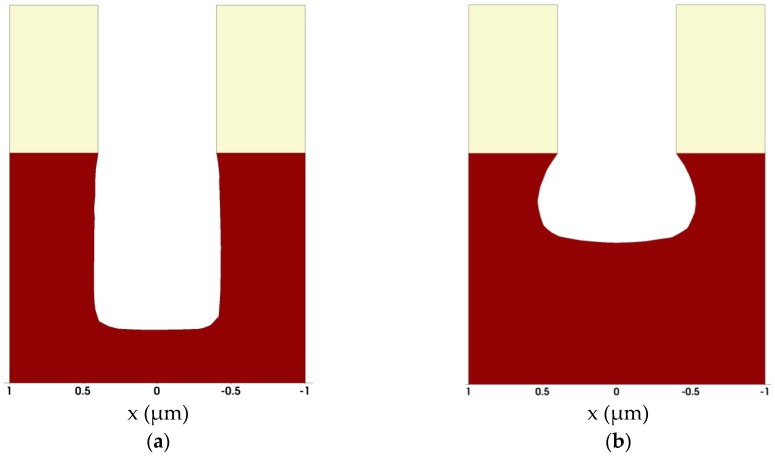
Example for the simulation of contact hole etching: (**a**) sputter etching with highly directional ion flux; (**b**) chemical dry etching with an isotropic angular distribution of the etching species. The material to be etched (for instance oxide) is shown in dark red. The mask which is assumed to be not affected by the etching process is depicted in yellow.

**Figure 10:**
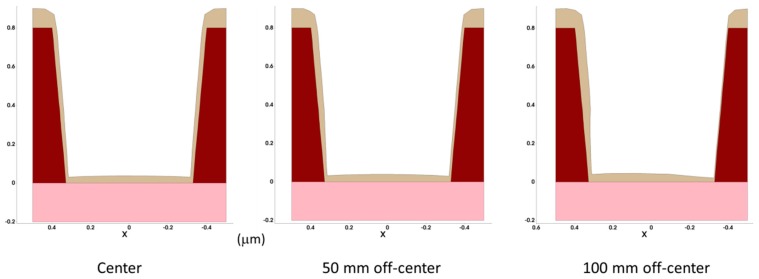
Example for the simulation of long-throw sputter deposition into a contact hole structure for different positions on the wafer. Ballistic transport of the metal atoms in the reactor is assumed, the target diameter and the distance between target and substrate were set to 300 mm and 150 mm, respectively. The step coverage (= ratio of the thickness at the sidewall bottom to the thickness on top) for the feature position at the center, 50 mm off-center, and 100 mm off-center is here given for sidewall left | sidewall right: 0.16 | 0.16, 0.16 | 0.12, and 0.20| 0.05, respectively.

DEP3D and ANETCH allow the simulation of these deposition and etching processes, provided that physical/chemical models suitable for the process in question have been implemented, parameters have been calibrated, and the required boundary conditions above the wafer are known, e.g., from equipment simulation. As an example, Figure 9 shows cross-sections of three-dimensional simulations of a contact hole etching process for two different angular distributions of the rate determining species: Figure 9a shows the result for purely physical sputter etching with a highly directional ion flux, Figure 9b depicts the result for chemical dry etching, that is, for an isotropic angular distribution of the etching species. Figure 10 shows simulation results for long-throw sputter deposition into a contact hole structure for different positions on the wafer. The asymmetry of the deposited layer for the off-axis positions of the contact hole is clearly visible.

#### 3.1.3. Integrated Topography Simulation

**Figure 11:**
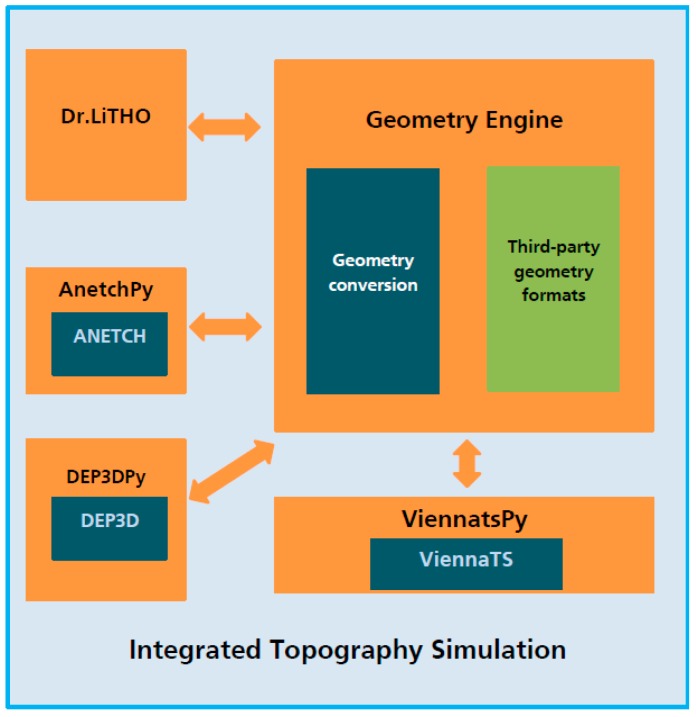
Integrated 3D topography simulation, combining Dr.LiTHO, DEP3D, ANETCH, and ViennaTS.

As outlined above, advanced nanoscale devices generally employ truly three-dimensional geometries. Furthermore, the approximation of these structures using rectangular shapes is no more possible, because the electrical performance depends on details, such as taper angles of FinFETs or corner rounding. Moreover, as discussed above already basic features such as line width depend on the three-dimensional interim structures generated during the patterning flow, e.g., in the form of non-vertical and/or non-straight resist edges. In turn, one of the key tasks in the SUPERAID7 project has been the development of an integrated three-dimensional topography simulator, which combines Dr.LiTHO [15], ANETCH [16], DEP3D [17], which all output a triangle-based surface representation, and the level-set based etching and deposition simulator ViennaTS [18]. To this end, DEP3D and ANETCH have been seamlessly integrated into the Python [29] simulation framework of Dr.LITHO. The ViennaTS level-set-method-based tool has been exposed to the Python programming language to allow the usage as a Python module. A common geometry conversion engine has been developed to handle the different data representations used in the tools, including surface and volume meshes, structured and unstructured grids. The physical models from DEP3D, ANETCH and ViennaTS are available in the integrated topography simulator and enable the simulation of a large variety of deposition and etching processes. Figure 11 shows the architecture of the integrated topography simulator.

**Figure 12:**
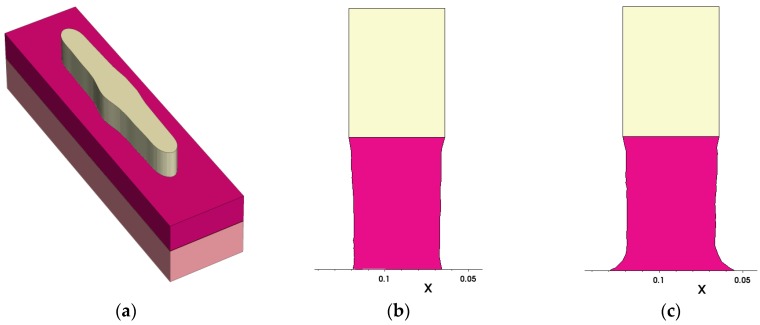
Coupled simulation of lithography and etching: (**a**) 3D simulation of the resist line geometry (yellow) above the polysilicon (purple); (**b**) simulated etched gate electrode for a position close to the center of the wafer (distance to center axis = 0.14 cm); (**c**) Simulated etched gate electrode for a position at the wafer rim (distance to center axis = 9.7 cm). The unit for the x axis in (**b**) and (**c**) is micron.

Figure 12 depicts an example for the combined simulation of lithography and etching: As layout, an SRAM cell pattern on polysilicon level has been used. The lithography simulation was performed with Dr.LiTHO assuming 193 nm water immersion with a strong off-axis illumination and unpolarized light. Dr.LiTHO provides the resist profile as a triangulated surface. To reduce the number of surface elements for the etching simulation, the footprint of the resist was extracted, smoothed and the 3D resist region was generated with steep sidewalls. Figure 12a shows one polysilicon line forming part of the SRAM layout. To study the effect of varying parameters on the etched profile, it is efficient to simulate etching of a slice located at the position where, according to the SRAM layout, the gate electrode located is above the active region. The etching simulations have been carried out with ANETCH using coupling to equipment simulation thus allowing one to study the effect of the feature position on the wafer. Details are provided elsewhere [28]. Briefly, an inductively-coupled plasma reactor is simulated, which is operated at a pressure of 1.5 Pa and powered with 1500 W, and the substrate is biased with 200 V. Cross sections of the etched gate electrode for two different positions on the wafer are shown in Figure 12b,c respectively. It can be seen that the shape particularly at the foot of the gate electrode significantly depends on the position on the wafer.

### 3.2. Integration with other Tools

In terms of process simulation and systematic process variations, the SUPERAID7 project has focused on topography simulation and the variations affecting the topography processes lithography, etching, and deposition: These processes and variations raise new challenges especially for three-dimensional nanoscale transistors as addressed in SUPERAID7 and in this paper.

For the simulation of the other process steps, especially ion implantation and dopant activation/diffusion, the well-established process simulator Sentaurus Process [13] has been used. To this end, results from SUPERAID7 topography simulation are transferred to Sentaurus Process via the DF-ISE file format of Synopsys. More specifically, the exported results are used by Sentaurus Process to update an initial structure by means of Boolean operations. Device simulation has been carried out with Sentaurus Device [13], and statistical device simulation with Garand of GSS (now belonging to Synopsys). To this end the SUPERAID7 topography simulation tools have been integrated with Sentaurus Process, the Sentaurus Workbench, and the statistical device simulator Garand. This overall hierarchical approach implemented in SUPERAID7 to simultaneously simulate the impact of systematic and stochastic variations on devices and circuits has been discussed elsewhere [30].

### 3.3. Compact Model Extraction

Because conventional compact models are not suitable for the three-dimensional devices as studied in SUPERAID7, a novel compact model (LETI-NSP) based on surface potential has been developed by CEA/Leti and used for the compact model extraction work in SUPERAID7. This model has among others been presented at IEDM 2016 [31].

**Figure 13:**
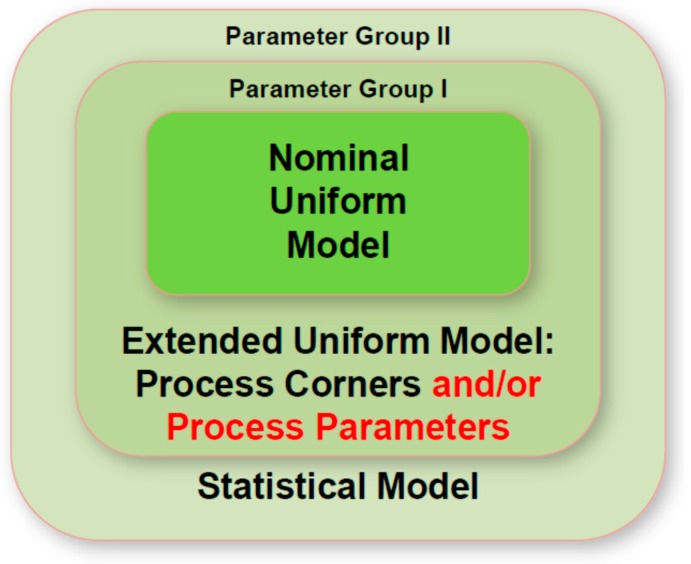
Extraction and generation of hierarchical compact model aware of systematic and statistical process variations.

Within SUPERAID7 the hierarchical approach developed at GSS (now Synopsys) to extract statistical compact models [8] has been used and extended. In the traditional approach illustrated in Figure 13 (without the red extension “and/or process parameters”), first a so-called “uniform” compact model is extracted from device simulations without taking process variations into account. Then, process corners are defined for the process results (e.g., channel length L and width W) generated by the systematic variations (e.g., focus F and dose D in a lithography step) to be considered. Following this, an extended compact model is extracted from device simulations at these process corners, using a first group of compact model parameters defined during this extraction. Finally, this compact model is further extended into a statistical compact model, extracted from statistical device simulations carried out at each of the process corners. This compact model then allows one to calculate for relevant electrical device data A (e.g., the threshold voltage or the leakage current) the probability distribution P(A,s) that certain values of A occur. This distribution depends on values s (e.g., L and W) of the process results generated by the systematic variations of process input (or equipment) data r (e.g., F and D) considered in the study. In order to get the final distribution of A, process simulations are carried out to calculate the dependence s(r) of the process results on the (equipment) parameter r. The final distribution P_A_ of the electrical device data is then given as:

P_A_ = ∫ P(A,s(r)) · f(r) dr (1)

where f(r) is the probability distribution of the (equipment) parameter r, e.g., the distributions of focus F and dose D.

Here, in general A is a vector, because various electrical properties are considered. As soon as more than one statistical variation occurs, also r is a vector. It should be noted that in case of more than one statistical variation, the components of r should be statistically independent. Otherwise it would be necessary to find the root cause of their dependence, and express this by relating them to some (hidden) parameters which cause the (partial) correlation.

Since one statistical variation may influence several process results (e.g., the channel length of one transistor and the channel width of another), s(r) is generally a matrix. This also allows the proper description of correlations, which arise in the case that an input parameter r influences more than one (intermediate or final) result parameter. The same holds for P(A,s).

In examples studied earlier (e.g., in [8]), process corners referred to the minimum and maximum values of two or three discrete parameters affected by systematic process variations, such as channel length and width, influenced among others by focus and dose variations in lithography. However, for three-dimensional nanoscale devices systematic process variations might not only lead to variations of such discrete parameters, but also change the shape of the device, e.g., the channel cross section. Such variations can hardly be described with the variation of just one or two geometrical parameters. In turn, the hierarchical compact model extraction strategy has been extended in SUPERAID7 as illustrated in Figure 13 (red text): The extended uniform compact model is not only based on process corners (e.g., channel length and width), but may also be based on the minimum and maximum values of statistically varying process parameters (e.g., focus F and dose D in lithography). In turn, compact model extraction is then not based only on the results of device simulation, but also on the result of coupled process and device simulation, needed in order to trace the impact of the parameter r on the device properties. If the extended uniform compact model is based on statistically varying process parameters r only, Equation (1) will simplify to:

P_A_ = ∫ P(A,r) · f(r) dr (2)

The mixed case where both process corners s and statistically varying process parameters r are used in the extraction of the extended compact model must be treated with some care, because only variables can be used in the compact model extraction which do not depend on each other and can therefore be treated independently. In turn, it must be differentiated between varying process parameters r_1_ which influence process corners s, and varying process parameters r_2_ which do not influence process corners s. The probability distribution of an electrical device performance parameter A is then given by

P_A_ = ∫ P(A,s(r_1_), r_2_) · f_1_(r_1_) · f_2_(r_2_) dr_1_ dr_2_ (3)

where f_1_(r_1_) and f_2_(r_2_) are the probability distributions of the varying process parameters r_1_ and r_2_, respectively.

The selection of the process corners and/or the varying process parameters to be considered in the extraction of the group I parameters (see Figure 13) depends on an a-priori assessment of the relevant variations. This assessment can be based on technological knowledge on which variations are most important, and/or on numerical simulation where each potentially relevant variation is considered alone. Then, the most relevant systematical variations are identified and used in the compact extraction process outlined above.

Generally, several different systematic variations may influence a device. Tracing all their combinations in parallel through the full simulation of the process sequence would require far too much effort. In turn, suitable design-of-experiment approaches must be used to limit the number of simulation splits. A simple and efficient approach is to first disregard variations which are due to expert knowledge already known to have negligible impact. Next, the impact of the remaining variations should be considered one by one, to identify a small number of most important variations. These variations could then either be considered in parallel, or more refined design-of experiment techniques could be employed to skip some or several elements of the full matrix of simulation splits.

### 3.4. Example for Compact Model Extraction

**Figure 14:**
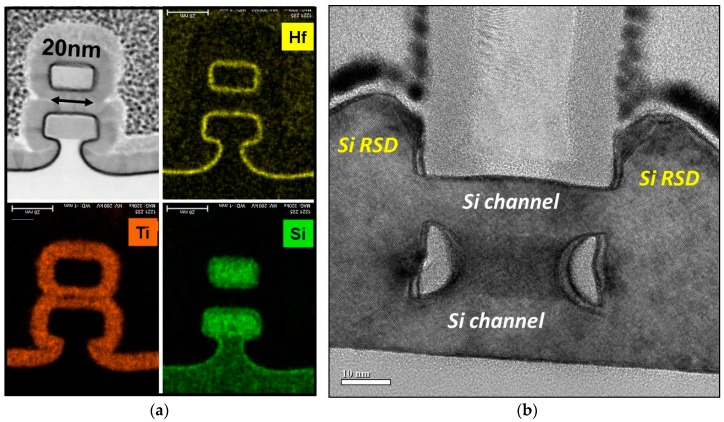
Example of nanowire transistors considered in this work: (**a**) cross section, including identification of Hf, Ti, and Si layers; (**b**) cut along source-drain direction, with SiN inner spacers.

Figure 14 shows a TEM micrograph of a nanowire transistor investigated in the benchmarks carried out in the SUPERAID7 project. The process flow included among others the deposition of a sacrificial SiGe layer on top of an SOI substrate, plus an upper Si layer. Self-Aligned Double patterning was used to create Si/SiGe/Si fins, and optical lithography for gate patterning. For this device architecture and the process flow used, ten potentially relevant systematical variations were identified. Their relative impact on the transistor performance was studied with coupled process and device simulation. Among these parameters, the three most important ones were identified [30]: (1) The SiGe mole fraction x_Ge_ which influences the etching of the inner spacers; (2) the fin SADP deposition factor d_sadp_ which gives the relative variation of the deposition thickness from the nominal thickness in the SADP spacer creation process; (3) the defocus F_Gate_ of the lithography step used to pattern the gate. Both for n-type NMOS and p-type PMOS MOSFETs, F_gate_ had the largest impact on both saturation current and leakage current.

**Figure 15:**
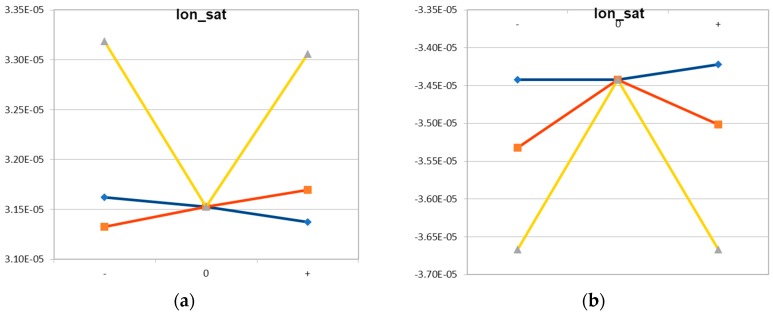
Example for extraction of extended compact model: Results for saturation current of (**a**) NMOS; (**b**) PMOS. Key: blue—x_Ge_; orange—d_sadp_; yellow—F_gate_. The horizontal axis shows the minimum, the nominal and the maximum value of the varying parameters.

**Figure 16:**
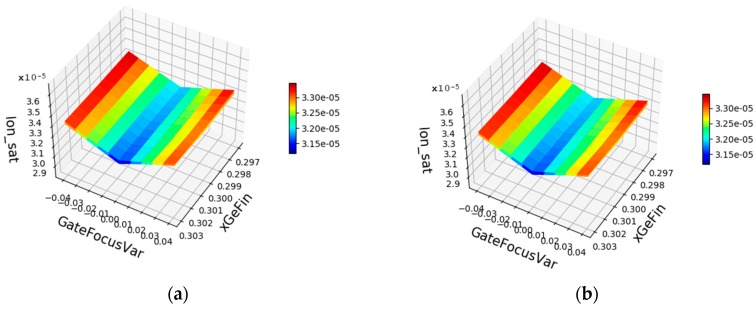

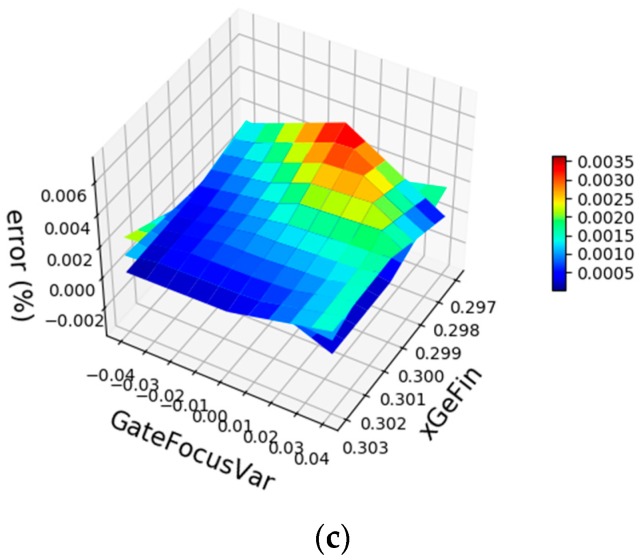
Example for extraction of extended compact model: Comparison between results (**a**) from TCAD; (**b**) from the extended compact model for the saturation current (color scale in Ampere of a nanowire NMOS; (**c**) relative error (color scale in %) of the compact model compared with TCAD.

Coupled process and device simulation was then carried out for the process corners of these three statistical variations. Figure 15 shows as an example the impact of these variations on NMOS and PMOS, respectively. The horizontal axis shows the minimum, the nominal and the maximum value of the varying parameters. Except for the parameter studied for each of the curves, the parameters have their nominal values. For instance, for the blue curve in Figure 15, x_Ge_ is varied whereas d_sadp_ and F_gate_ have their nominal values. The V-shape for the impact of F_gate_ is an intrinsic feature of the dependence of CD on defocus in lithography, discussed above in connection with Figure 2. For d_sadp_ a technologically appropriate variation was assumed. Its impact was, however, smaller than the impact of numerical discretization errors. In turn, in further work, a larger variation will be assumed here for the extraction of the compact model, whereas the realistic value can and will then be introduced via the probability distribution f(d_sadp_) in Equation (2). Based on these variations, the extended compact model was extracted, using the Mystic [13] tool from Synopsys. Figure 16 shows as an example the comparison between the saturation current simulated by TCAD and the results from the extended compact model extracted. Further work includes among others the updated extraction of the dependence on the variations of X_Ge_ and the final extraction of the statistical compact model, as indicated in Figure 13. The result will be reported elsewhere.

## 4. Discussion

Especially for three-dimensional nanoscale transistors, the details of the geometries generated during device fabrication are important both for the nominal device performance and for its stability against systematic variations caused by process equipment and/or by layout effects. Other than for statistical variations which can be simulated with stand-alone statistical device simulation, there is a huge variety of potential systematic variations, which must be addressed with various equipment simulation tools and process simulation modules. In turn, it is indispensable for optimized device and circuit manufacturing to well identify and characterize the sources of systematic variability, to sort out those with the largest impact, depending on the device and circuit in question, and to quantify their joint impact on the final nanoelectronic product. The impacts of systematic and statistical variability must be simulated and minimized in parallel, by adapting process flows, but potentially also device architectures and even fabrication equipment. In this paper, a holistic approach to use coupled equipment, process and device simulation combined with the extraction of variation-aware compact models has been presented together with some examples. The approach is partly based on well-established tools used in industry, partly on additional modules and extensions which are compatible to such standard frameworks. The suggestions made are intended to support industry in their efforts to optimize the stability of their products against all kinds of process variations.
